# Secondary Metabolites from the Nematode-Trapping Fungus *Dactylellina haptotyla* YMF1.03409

**DOI:** 10.3390/microorganisms11112693

**Published:** 2023-11-03

**Authors:** Hongmei Lei, Guangke Zhang, Peiji Zhao, Guohong Li

**Affiliations:** State Key Laboratory for Conservation and Utilization of Bio-Resources in Yunnan, School of Life Sciences, Yunnan University, Kunming 650091, China

**Keywords:** nematode-trapping fungus, *Dactylellina haptotyla* YMF1.03409, traps, secondary metabolites, nematicidal

## Abstract

As a representative nematode-trapping fungus, *Dactylellina haptotyla* can capture and kill nematodes by producing traps, known as adhesive knobs. In this paper, the strain of *D. haptotyla* YMF1.03409 was studied by means of medium screening, fermentation, and purification and identification of crude extracts. Eighteen compounds were obtained from *D. haptotyla* YMF1.03409, including two new metabolites, nosporins C (**1**) and D (**2**). The known metabolites were identified to be 3-chloro-4-methoxybenzaldehyde (**3**), 3-chloro-4-methoxybenzoic acid (**4**), 2-chloro-1-methoxy-4-(methoxymethyl)benzene (**5**), 3-hydroxy-3-methyloxindole (**6**), nicotinic acid (**7**), succinic acid (**8**), 3,4-dihydroxybutanoic acid (**9**), 5′-O-methyladenosine (**10**), uridine (**11**), 2′-deoxyuridine (**12**), thymidine (**13**), 3-(phenylmethyl)-2,5-morpholinedione (**14**), methyl-β-D-glucopyranoside (**15**), 1,2-benzenedicarboxylic acid bis(2-methyl heptyl) ester (**16**), β-sitosterol (**17**), and 3β,6α-diol-stigmastane (**18**). The bioactive assay showed that these compounds had no obvious nematicidal activity against the nematodes *Meloidogyne incognita* and *Panagrellus redivivus*.

## 1. Introduction

Plant pathogenic nematodes can wreak havoc on crop productivity [1]. Root-knot nematodes are the most prominent pathogenic nematodes, which have numerous hosts and a wide distribution. Their quick reproduction, environmental adaptability, and ease of dissemination make them challenging to control. The current methods for controlling plant pathogenic nematodes mainly include chemical control, agricultural control, and biological control. Chemical control has the advantages of short cycle and quick effect, and plays an important role in ensuring a high and stable yield of crops [2]. Despite their efficiency, chemical pesticides can easily contaminate the environment. Pesticide residues that are left on agricultural products have the potential to harm soil structure, create soil erosion, and pose a concern to food safety [3]. Agricultural control conquers nematode infestation mainly via crop rotation, flooding, and culturing anti-nematode plants. These methods may be time-consuming and incomplete in their control of pathogenic nematodes [4].

Due to the aforementioned disadvantages, biological control has been widely emphasized in recent years. Research on the development of biological control agents using nematophagous microbial resources, such as nematode-trapping fungi, has emerged as a popular topic in the control of nematodes [3]. Nematode-trapping fungi constitute a specialized group of fungi that can catch and kill nematodes by producing traps [5]. *Dactylellina haptotyla* is a canonical model of nematode-trapping fungi, and current research on the species has mainly focused on the physiological mechanisms underlying how it controls nematodes, but research on its secondary metabolites is rarely reported [6,7,8]. In the present study, eighteen compounds were obtained from the strain *D. haptotyla* YMF1.03409, and their structural types were found to mainly include polyketides, aromatics, and nucleosides.

## 2. Materials and Methods

### 2.1. Experimental Strain and Culture

*D. haptotyla* YMF1.03409 was isolated from soil in Longquan Forest Garden of Yunnan Yuxi Yimen County, which was preserved in the State Key Laboratory of Conservation and Utilization of Biological Resources in Yunnan, China. *D. haptotyla* YMF1.03409 was routinely cultured on potato dextrose agar (PDA) plates at 28 °C for 21 days.

The nematodes used in this study were *Panagrellus redivivus* and *Meloidogyne incogntta*. *P. redivivus* was cultured in an oat medium at 25 °C. *M. incognita* was obtained from infected tomato roots in Yunnan Yuanmou County.

The following two steps were involved in the acquisition of *M. incognita*: (1) nematode egg masses were collected from the roots of infested plants and incubated in double-distilled water at 25 °C for three days, and (2) the collection of nematodes was achieved by centrifuging the sample at 3000 rpm for three minutes.

### 2.2. General Experimental Procedures

Precoated silica gel GF254 plates (Qingdao Marine Chemical Inc., Shandong, China) with various solvent systems were used for thin-layer chromatography (TLC). Column chromatography was carried out utilizing Sephadex LH-20 (Amersham Biosciences, Uppsala, Sweden) and silica gel (Qingdao Marine Chemical Inc., Shandong, China). The ultraviolet-visible (UV) spectrum was recorded using a Shimadzu UV-2401PC spectrophotometer (Shimadzu, Tokyo, Japan), and the λmax (log ε) value was reported in nm. Nuclear magnetic resonance (NMR) analysis was performed using a Bruker Avance III 600 NMR spectrometer (Bruker, MA, USA) with tetramethylsilane (TMS) as an internal standard. High-resolution electrospray ionization mass spectroscopy (HR-ESI-MS) and ESI-MS data were detected and recorded using a VG Auto-Spec-3000 mass spectrometer (VG, Manchester, UK). A Jasco DIP-370 digital polarimeter (JASCO, Tokyo, Japan) was used to determine and collect the optical rotation data of the compounds.

### 2.3. Screening of Culture Conditions

*D. haptotyla* YMF1.03409 was inoculated in the nine media listed in Table 1. The culture volume was 300 mL, and the solid-state culture conditions (#5, #6, #7, #8, and #9) were 28 °C for 31 days. The liquid-state culture conditions (#1, #2, #3, and #4) were 28 °C at 180 rpm for 14 days. After the completion of the culture, the fermentation products were processed as follows: the liquid culture products were filtered through eight layers of gauze to remove mycelia; equivalent amounts of ethyl acetate were used to extract the fermentation broth; and the extracts were combined and evaporated under reduced pressure on a rotary evaporator to obtain the crude extracts. The solid culture products were pounded into small pieces and extracted using ethyl acetate/methanol/glacial acetic acid (80:15:5, *v*/*v*/*v*/*v*) for three, two, and one days, respectively, and finally, the soaking solutions were combined and evaporated under reduced pressure to obtain the crude extracts. These crude extracts were then weighted.

### 2.4. Fermentation and Isolation of Compounds

Medium #9 was selected to culture *D. haptotyla* YMF1.03409 at 28 °C for 31 days in a total volume of 40 L. The solid fermentation products were cut into small pieces and extracted exhaustively using the mixture solution (ethyl acetate/methanol/acetic acid = 80:15:5, *v*/*v*/*v*) six times. The filtrate was evaporated using a rotary evaporator under reduced pressure to obtain the crude extracts (35.64 g).

The crude extract was eluted using a silica gel column (200–300 mesh) with petroleum ether/ethyl acetate (20:1→7:3, *v*/*v*), chloroform/methanol (20:1→7:3, *v*/*v*), and methanol to yield twelve fractions (Fr.1–12). Fr.3 (2.085 g) was further separated using a column of silica gel (200–300 mesh) via elution with petroleum ether/ethyl acetate (50:1→8:2, *v*/*v*) to obtain eleven fractions (Fr.3.1–11). Fr.3.1 (731 mg) was purified using a column of silica gel (200–300 mesh) via elution with petroleum ether/acetone (50:1→8:2, *v*/*v*) to yield nine fractions (Fr.3.1.1–9). Fr.3.1.7 (20 mg) was further loaded on a Sephadex LH-20 and eluted with acetone to obtain **5** (3 mg). Fr.3.2 (400 mg) was separated using a Sephadex LH-20 via elution with acetone and further purified using a silica gel column to yield **3** (2 mg) and **4** (15 mg). Fr.3.5 (205 mg) was chromatographed over a silica gel column (200–300 mesh, 40 g) using petroleum ether/ethyl acetate (50:1→8:2) to yield eight fractions (Fr.3.5.1–8). Fr.3.5.6 (190 mg) was loaded on a silica gel column (200–300 mesh, 40 g) and eluted with petroleum ether/acetone (200:1→10:1) to produce three fractions (Fr.3.5.6.1–3). Fr.3.5.6.3 (172 mg) was purified using a Sephadex LH-20 column via elution with acetone to yield **17** (22 mg).

Fr.8 (3.167 g) was purified using a chloroform/methanol (1:1, *v*/*v*) gel column to yield thirteen fractions (Fr.8.1–13). Fr.8.4 (389 mg) was chromatographed over a silica gel column (200–300 mesh) and eluted with petroleum ether/acetone (50:1→8:2, *v*/*v*) to produce eleven fractions (Fr.8.4.1–11). Fr.8.4.2 (13 mg) was separated using a Sephadex LH-20 via elution with methanol to produce **6** (2 mg). Fr.8.7 (796 mg) was separated using a silica gel column (200–300 mesh) with chloroform/methanol (100:1→8:2, *v*/*v*) to give twelve fractions (Fr.8.7.1–12). Fr.8.7.12 (157 mg) was purified using a Sephadex LH-20 (methanol) to obtain **9** (2 mg). Fr.8.8 (147 mg) was separated using a silica gel column (200–300 mesh) with petroleum ether/ethyl acetate (50:1→8:2, *v*/*v*) to give seven fractions (Fr.8.8.1–7). Fr.8.8.6 (55 mg) was loaded on a silica gel column (200–300 mesh) with chloroform/methanol (100:1→8:2, *v*/*v*) and then purified using a Sephadex LH-20 (methanol) to produce **8** (27 mg). 

Fr.9 (428 mg) was purified using a Sephadex LH-20 via elution with methanol to obtain seven fractions (Fr.9.1–7). Fr.9.3 (74 mg) was chromatographed over a silica gel column (200–300 mesh) and eluted with chloroform/methanol (200:1→10:1, *v*/*v*) to produce nine fractions (Fr.9.3.1–9). Fr.9.3.4 (4 mg) was further loaded on a Sephadex LH-20 and eluted with methanol to obtain **18** (1 mg). Fr.9.5 (184 mg) was chromatographed over a silica gel column (200–300 mesh) and eluted with chloroform/methanol (50:1→8:2, *v*/*v*) to produce seven fractions (Fr.9.5.1–7). Fr.9.5.7 (40 mg) was further purified using a Sephadex LH-20 via elution with methanol to obtain **7** (1 mg).

Fr.10 (3 g) was purified using a Sephadex LH-20 via elution with methanol to obtain five fractions (Fr.10.1–5). Fr.10.3 (380 mg) was chromatographed over a silica gel column (200–300 mesh) and eluted with chloroform/acetone (50:1→8:2, *v*/*v*) to produce seven fractions (Fr.10.3.1–7). Fr.10.3.5 (74 mg) was separated using a Sephadex LH-20 via elution with methanol to yield **1** (1 mg) and **2** (4 mg). Fr.10.3.7 (113 mg) was separated using a silica gel column (200–300 mesh) with chloroform/methanol (50:1→7:3, *v*/*v*) to give three fractions (Fr.10.3.7.1–3). Fr.10.3.7.3 (6 mg) was separated using a Sephadex LH-20 (acetone) to yield **13** (1 mg). Fr.10.4 (557 mg) was purified using a column of silica gel (200–300 mesh) and eluted with chloroform/methanol (50:1→7:3, *v*/*v*) to obtain **14** (5 mg). Fr.10.3.4 (83 mg) was purified using a Sephadex LH-20 via elution with methanol to yield **16** (1 mg).

Fr.11 (1.431 g) was purified using a Sephadex LH-20 via elution with methanol to obtain seven fractions (Fr.11.1–7). Fr.11.5 (729 mg) was chromatographed over a silica gel column (200–300 mesh) and eluted with chloroform/methanol (50:1→8:2, *v*/*v*) to produce 11 fractions (Fr.11.5.1–11). Fr.11.5.7 was separated using a Sephadex LH-20 (methanol) to yield **10** (2 mg). Fr.11.5.9 (30 mg) was chromatographed over a silica gel column (200–300 mesh) and eluted with chloroform/methanol (100:1→8:2, *v*/*v*) to produce four fractions (Fr.11.5.9.1.1–4). Fr.11.5.9.2 (5 mg) was purified using a Sephadex LH-20 (methanol) to obtain **12** (1 mg). Fr.11.5.11 (200 mg) was chromatographed over a silica gel column (200–300 mesh) and eluted with ethyl acetate/methanol (100:1→8:2, *v*/*v*) to produce three fractions (Fr.11.5.11.1–3). Fr.11.5.11.1 (132 mg) was chromatographed over a Sephadex LH-20 (methanol) to yield **11** (2 mg) and **15** (18 mg). 

### 2.5. Nematicidal Activity of Compounds

The nematicidal activity test consisted of the following steps: (1) the compounds were dissolved in a methanol–water solution that contained 3% methanol, and the concentration of the compounds was measured at 400 ppm. As a control, the methanol–water solution that contained 3% methanol was used. (2) About 150 juveniles (J2) of *M. incognita* or *P. redivivus* were transferred to 3.5 cm Petri dishes containing either the compound solution or the control solution. (3) The assay was performed in triplicate, and each replicate was performed with three Petri dishes. The numbers of dead and live nematodes were counted after 12, 24, and 48 h using a light microscope (Olympus, Tokyo, Japan) [9].

## 3. Results

### 3.1. Culture and Fermentation of D. haptotyla YMF1.03409

**Figure 1:**
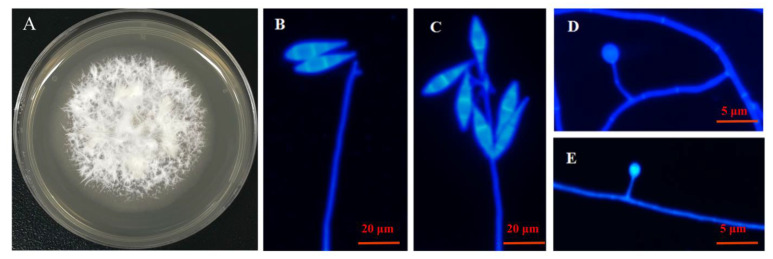
The characteristics of *D. haptotyla* YMF1.03409. (**A**) The colony status of *D. haptotyla* YMF1.03409 grown on potato dextrose agar (PDA) for 21 days. (**B**,**C**) The conidiophores of *D. haptotyla* YMF1.03409 (**D**,**E**) the adhesive knob of *D. haptotyla* YMF1.03409.

*D. haptotyla* is one of the representative nematode-trapping fungi (Figure 1A), which belongs to the genus *Dactylellina* of the family Orbiliaceae (Ascomycota). Its conidiospores are fusiform or teardrop-shaped (Figure 1B,C). Its trap is an adhesive knob, which is ovoid and grows on vegetative mycelia (Figure 1D,E). 

**Figure 2:**
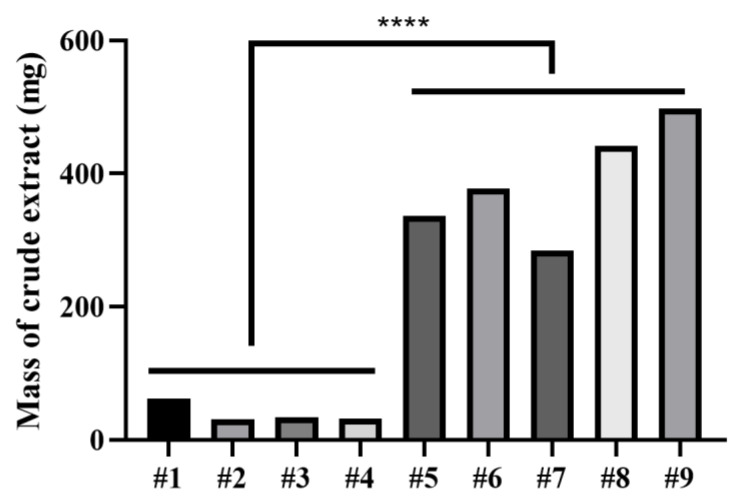
Mass of crude extracts under nine culture conditions. The horizontal coordinates represent the numbers of the nine culture conditions. **** represents *p*-value < 0.0001, which was calculated using the Student’s *t*-test.

The optimal fermentation conditions were determined based on the mass of crude extracts. Firstly, by comparing the mass of crude extracts under different conditions, it was found that when rice was included in the media’s composition (#5, #6, #7, #8, and #9), the mass of crude extracts under the corresponding culture conditions was significantly higher than when rice was not included (#1, #2, #3, and #4). Detailed information on the mass of crude extracts under the corresponding culture conditions is shown in Figure 2.

Among the media #5, #6, #7, #8, and #9, medium #9 showed the highest mass of crude extracts; thus, medium #9 was finally determined as the amplified fermentation medium, and the culture condition was 28 °C for 31 days of static incubation.

### 3.2. Structural Identification of Compounds

Medium #9 was selected to culture *D. haptotyla* YMF1.03409. Eighteen compounds were isolated from the crude extracts. Their structures were identified based on the obtained NMR and MS data.

Compound **1:** This compound is a white solid, with ESI-MS: 251 [M + Na]^+^; HR-ESI-MS: 251.0524 ([M + Na]^+^); [α]${}_{D}^{20}$ = 11.5 (*c* = 0.10, MeOH); and UV (MeOH) λ_max_ (log ε) nm: 202 (3.16), 221 (2.81), and 277 (2.79).

An analysis of the HR-ESI-MS data revealed a molecular formula of C_10_H_12_O_6_ based on the [M + Na]^+^ ion signal at *m*/*z* 251.0524 (calcd. for C_10_H_12_O_6_Na, 251.0526). The spectroscopic data (Table 2) of compound **1** are basically the same as those of nosporin A, except that the methyl group at 9-OH is changed to a formate group in compound **1 [10]**.

**Figure 3:**
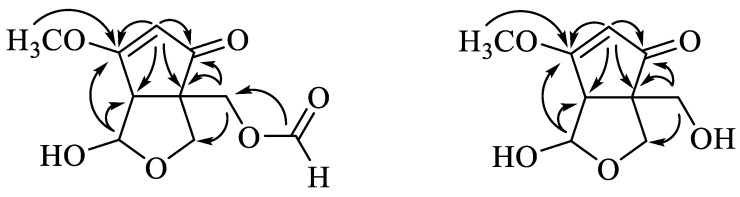
Selected ^1^H detected heteronuclear multiple bond correlations (HMBC) of **1** and **2**.

**Figure 4:**
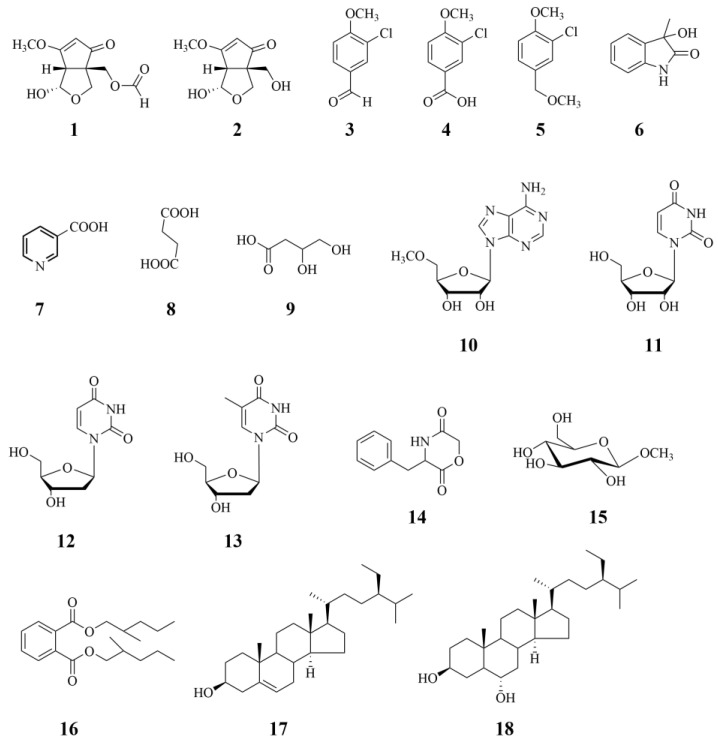
Structures of compounds from *D. haptotyla* YMF1.03409.

This deduction was confirmed by the 2D-NMR experiment (Figure 3): H-2 (δ_H_ 5.35) correlated with the carbons C-1 (δ_C_ 203.4), C-3 (δ_C_ 187.8), C-4 (δ_C_ 56.9), and C-5 (δ_C_ 58.3); H-4 (δ_H_ 3.28) correlated with the carbons C-1 (δ_C_ 203.4), C-2 (δ_C_ 105.1), C-3 (δ_C_ 187.8), C-8 (δ_C_ 98.4), and C-9 (δ_C_ 62.9); H-6 (δ_H_ 3.92 and 4.06) correlated with the carbons C-1 (δ_C_ 203.4) and C-9 (δ_C_ 62.9); H-8 (δ_H_ 5.59) correlated with the carbons C-6 (δ_C_ 69.5), C-3 (δ_C_ 187.8), and C-5 (δ_C_ 58.3); H-9 (δ_H_ 4.34 and 4.54) correlated with the carbons C-1 (δ_C_ 203.4), C-6 (δ_C_ 69.5), C-5 (δ_C_ 58.3), and C-4 (δ_C_ 56.9); 9-OCHO (δ_H_ 8.03) correlated with the carbon C-9 (δ_C_ 62.9); and 3-OCH_3_ (δ_H_ 3.90) with the carbon C-3 (δ_C_ 187.8). The relative configuration of **1** was assigned on the basis of nuclear Overhauser effect spectroscopy (NOESY) correlations between H-4 and H-9 and H-8 (Figure 3). Based on the data presented above, compound **1** was identified as nosporin C (Figure 4).

Compound **2:** This compound is a white solid with ESI-MS: 223 [M + Na]^+^; HR-ESI-MS: 223.0576 ([M + Na]^+^); [α]${}_{D}^{20}$= 6.2 (*c* = 0.10, MeOH); and UV (MeOH) λmax (log ε) nm: 200 (3.55), 221 (2.89), and 277 (2.61).

An analysis of the HR-ESI-MS data revealed a molecular formula of C_9_H_12_O_5_ based on the [M + Na]^+^ ion signal at *m*/*z* 223.0576 (calcd. for C_9_H_12_O_5_Na, 233.0577). An analysis of the spectroscopic data (Table 2) revealed that compound **2** is similar to compound **1** and the methyl group at 9-OH is not replaced (Figure 4). A 2D-NMR experiment verified this deduction (Figure 3). The relative configuration of **2** was determined via NOESY correlations between H-4 and H-9 and H-8. Based on the data presented above, compound **2** was identified as nosporin D (Figure 4).

Compound **3:** Colorless oil, the molecular formula of compound **3** is C_8_H_7_ClO_2_. ESI-MS: 193 [M + Na]^+^; ^1^H-NMR (CDCl_3_, 600 MHz) δ_H_: 4.00 (3H, s), 7.05 (1H, d, *J* = 8.5 Hz), 7.79 (1H, dd, *J* = 2.0, 8.5 Hz), 7.91 (1H, d, *J* = 2.0 Hz); ^13^C-NMR (CDCl_3_, 150 MHz) δ_C_: 56.5 (q), 111.7 (d), 123.7 (s), 130.3 (s), 130.5 (d), 131.2 (d), 159.8 (s), 189.7 (d). It was identified as 3-chloro-4-methoxybenzaldehyde based on the data of reference [11].

Compound **4:** Colorless oil, the molecular formula of compound **4** is C_8_H_7_ClO_3_. ESI-MS: 209 [M + Na]^+^; ^1^H-NMR (CD_3_OD, 600 MHz) δ_H_: 3.95 (3H, s), 7.15 (1H, d, *J* = 8.6 Hz), 7.97 (1H, dd, *J* = 1.9, 8.6 Hz), 7.90 (1H, d, *J* = 1.9 Hz); ^13^C-NMR (CD_3_OD, 150 MHz) δ_C_: 56.9 (q), 112.8 (d), 123.4 (s), 125.2 (s), 131.3 (d), 132.5 (d), 160.2 (s), 168.7 (s). It was identified as 3-chloro-4-methoxybenzoic acid based on the data of reference [11].

Compound **5:** Colorless oil, the molecular formula of compound **5** is C_9_H_11_ClO_2_. ESI-MS: 209 [M + Na]^+^; ^1^H-NMR (CDCl_3_, 600 MHz) δ_H_: 3.49 (3H, s), 3.90 (3H, s), 5.01 (2H, s), 6.91 (1H, d, *J* = 8.5 Hz), 7.25 (1H, dd, *J* = 2.0, 8.5 Hz), 7.39 (1H, d, *J* = 2.0 Hz); ^13^C-NMR (CDCl_3_, 150 MHz) δ_C_: 50.9 (q), 56.2 (q), 65.3 (t), 111.9 (d), 128.1 (d), 1129.1 (s), 130.5 (d), 159.8 (s). It was identified as 2-chloro-1-methoxy-4-(methoxymethyl)benzene (Figure 4) based on the data of reference [12].

Compound **6:** Colorless oil, the molecular formula of compound **6** is C_9_H_9_NO_2_. ESI-MS: 164 [M + H]^+^; ^1^H-NMR (CD_3_OD, 600 MHz) δ_H_: 1.48 (3H, s), 6.88 (1H, d, *J* = 7.7 Hz), 7.04 (1H, t, *J* = 7.6 Hz), 7.24 (1H, t, *J* = 7.7 Hz), 7.34 (1H, d, *J* = 7.4 Hz); ^13^C-NMR (CD_3_OD, 150 MHz) δ_C_: 24.7 (q), 74.7 (s), 111.2 (d), 123.7 (d), 124.5 (d), 130.4 (d), 134.4 (s), 142.1 (s), 182.6 (s). It was identified as 3-hydroxy-3-methyloxindole (Figure 4) based on the data of reference [13].

Compound **7:** Colorless oil, the molecular formula of compound **7** is C_6_H_5_NO_2_. ESI-MS: 124 [M + H]^+^; ^1^H-NMR (CD_3_OD, 600 MHz) δ_H_: 7.55 (1H, dd, *J* = 7.6, 5.0 Hz), 8.40 (1H, d, *J* = 8.0 Hz), 8.71 (1H, d, *J* = 5.9 Hz), 9.11 (1H, s); ^13^C-NMR (CD_3_OD, 150 MHz) δ_C_: 125.2 (d), 129.0 (s), 139.2 (d), 151.3 (d), 153.5 (d), 168.0 (s). It was identified as nicotinic acid (Figure 4) based on the data of reference [14].

Compound **8:** Colorless oil, the molecular formula of compound **8** is C_4_H_6_O_4_. ESI-MS: 119 [M + H]^+^; ^1^H-NMR (CD_3_OD, 600 MHz) δ_H_: 2.67 (4H, s); ^13^C-NMR (CD_3_OD, 150 MHz) δ_C_: 30.6 (t), 181.6 (s). It was identified as succinic acid (Figure 4) based on the data of reference [15].

Compound **9:** Colorless oil, the molecular formula of compound **9** is C_4_H_8_O_4_. ESI-MS: 121 [M + H]^+^; ^1^H-NMR (CD_3_OD, 600 MHz) δ_H_: 2.36 (1H, d, *J* = 17.7 Hz), 2.82 (1H, dd, 5.9, *J* = 17.7 Hz), 4.21 (1H, d, *J* = 10.0 Hz), 4.42 (1H, dd, *J* = 4.4, 10.0 Hz), 4.55 (1H, m); ^13^C-NMR (CD_3_OD, 150 MHz) δ_C_: 38.5 (t), 68.4 (d), 77.7 (d), 179.1 (s). It was identified as 3,4-dihydroxybutanoic acid (Figure 4) based on the data of reference [16].

Compound **10:** White solid, the molecular formula of compound **10** is C_10_H_13_N_5_O_4_. ESI-MS: 282 [M + H]^+^; ^1^H-NMR (CD_3_OD, 600 MHz) δ_H_: 3.19 (3H, s, 5′-OCH_3_), 3.65 (1H, dd, *J* = 2.4, 12.5 Hz, H-5a′), 3.78 (1H, dd, *J* = 2.6, 12.5 Hz, H-5b′), 4.05 (1H, m, H-4′), 4.232 (1H, m, H-3′), 4.38 (1H, m, H-2′), 5.95 (1H, d, *J =* 5.8 Hz, H-1′), 8.07 (1H, s, H-2′), 8.21 (1H, s, H-8); ^13^C-NMR (CD_3_OD, 150 MHz) δ_C_: 89.2 (d, C-1′), 84.6 (d, C-2′), 70.8 (d, C-3′), 88.4 (d, C-4′), 63.2 (t, C-5′), 58.8 (q, 5′-OCH_3_), 153.6 (d, C-2), 150.0 (s, C-4), 120.9 (s, C-5), 157.6 (s, C-6), 141.9 (d, C-8). It was identified as 5′-*O*-methyladenosine (Figure 4) based on the data of reference [17].

Compound **11:** White solid, the molecular formula of compound **11** is C_9_H_12_N_2_O_6_. ESI-MS: 267 [M + Na]^+^; ^1^H-NMR (CD_3_OD, 600 MHz) δ_H_: 8.01 (1H, d, *J* = 8.1 Hz, H-6), 5.89 (1H, d, *J* = 4.7 Hz, H-1′), 5.69 (1H, d, *J* = 8.1 Hz, H-5′), 4.18 (1H, m, H-2′), 4.16 (1H, m, H-3′), 4.00 (1H, m, H-4′), 3.84 (1H, dd, *J* = 2.6, 12.2 Hz, H-5a′), 3.73 (1H, dd, *J* = 3.1, 12.2 Hz, H-5b′). ^13^C-NMR (CD_3_OD, 150 MHz) δ_C_: 166.2 (C-4), 152.5 (C-2), 142.7 (C-6), 102.6 (C-5), 90.7 (C-1′), 86.4 (C-4′), 75.7 (C-3′), 71.3 (C-2′), 62.3 (C-5′). It was identified as uridine (Figure 4) based on the data of reference [18].

Compound **12:** White solid, the molecular formula of compound **12** is C_9_H_12_N_2_O_5_. ESI-MS: 251 [M + Na]^+^; ^1^H-NMR (CD_3_OD, 600 MHz) δ_H_: 7.98 (1H, d, *J* = 8.1 Hz), 6.27 (1H, t, *J* = 6.7 Hz, H-1′), 5.69 (1H, d, *J* = 8.1 Hz), 4.38 (1H, m, H-4′), 3.91 (1H, m, H-3′), 3.77 (1H, dd, *J* = 3.2, 12.0 Hz, H-5a′), 3.72 (1H, dd, *J* = 3.7, 12.0 Hz, H-5b′), 2.27 (1H, m, H-2a′), 2.22 (1H, m, H-2b′); ^13^C-NMR (CD_3_OD, 150 MHz) δ_C_: 166.0 (s, C-4), 152.2 (s, C-2), 142.5 (d, C-6), 102.6 (d, C-5), 89.0 (d, C-1′), 86.6 (d, C-4′), 72.3 (d, C-3′), 62.8 (t, C-5′), 41.4 (t, C-2′). It was identified as 2′-deoxyuridine (Figure 4) based on the data of reference [19].

Compound **13:** Colorless oil, the molecular formula of compound **13** is C_10_H_13_N_2_O_5_. ESI-MS: 265 [M + Na]^+^; ^1^H-NMR (CD_3_OD, 600 MHz) δ_H_: 7.81 (1H, brs, H-6), 6.28 (1H, t, *J* = 6.9 Hz, H-7), 4.40 (1H, t, *J* = 2.9 Hz, H-10), 3.90 (1H, brs, H-9), 3.80 (2H, m, H-12), 2.23 (2H, m, H-8), 1.87 (3H, s, 5-CH_3_); ^13^C-NMR (CD_3_OD, 150 MHz) δ_C_: 166.4 (s, C-4), 152.4 (s, C-2), 138.2 (d, C-6), 111.5 (s, C-5), 88.8 (d, C-7), 86.2 (d, C-10), 72.2 (d, C-9), 62.8 (t, C-12), 41.2 (t, C-8), 12.4 (q, 5-CH_3_). It was identified as thymidine (Figure 4) based on the data of reference [20].

Compound **14:** Colorless oil, the molecular formula of compound **14** is C_11_H_11_NO_3_. ESI-MS: 206 [M + H]^+^; ^1^H-NMR (CD_3_OD, 600 MHz) δ_H_: 7.46 (2H, m), 7.29–7.34 (3H, m), 4.55 (1H, brs), 4.41 (1H, d, *J* = 10.0 Hz), 4.21 (1H, d, *J* = 10.0 Hz), 2.82 (1H, dd, *J* = 17.7, 5.8 Hz), 2.36 (1H, d, *J* = 17.7 Hz); ^13^C-NMR (CD_3_OD, 150 MHz) δ_C_: 179.1 (s), 176.8 (s), 141.2 (s), 129.4 (d), 129.4 (d), 129.1 (d), 128.0 (d), 77.7 (t), 68.4 (d), 38.5 (t). It was identified as 3-(phenylmethyl)-2,5-morpholinedione (Figure 4) based on the data of reference [21].

Compound **15:** Colorless oil, the molecular formula of compound **15** is C_7_H_14_O_6_. ESI-MS: 217 [M + Na]^+^; ^1^H-NMR (CD_3_OD, 600 MHz) δ_H_: 3.16 (1H, m), 3.26 (1H, m), 3.30 (1H, m), 3.37 (1H, m), 3.52 (3H, s), 3.67 (1H, dd, *J* = 12.1, 4.6 Hz), 3.87 (1H, dd, *J* = 12.4, 2.5 Hz), 4.16 (1H, d, *J* = 7.8 Hz); ^13^C-NMR (CD_3_OD, 150 MHz) δ_C_: 105.4 (d), 78.0 (s), 77.9 (d), 75.0 (d), 71.6 (d), 62.7 (t), 57.3 (q). It was identified as methyl-β-D-glucopyranoside (Figure 4) based on the data of reference [22].

Compound **16:** Colorless oil, the molecular formula of compound **16** is C_20_H_30_O_4_. ESI-MS: 413 [M + Na]^+^; ^1^H-NMR (CD_3_OD, 600 MHz) δ_H_: 7.55 (1H, dd, *J* = 7.6, 5.0 Hz), 8.40 (1H, d, *J* = 8.0 Hz), 8.71 (1H, d, *J* = 5.9 Hz), 9.11 (1H, s); ^13^C-NMR (CD_3_OD, 150 MHz) δ_C_: 169.3 (s), 133.6 (s), 132.4 (d), 129.9 (d), 69.1 (t), 40.2 (d), 31.6 (t), 30.1 (t), 25.0 (t), 24.0 (t), 14.0 (q), 11.4 (q). It was identified as 1,2-benzenedicarboxylic acid bis(2-methyl heptyl) ester (Figure 4) based on the data of reference [23].

Compound **17:** Colorless oil, the molecular formula of compound **17** is C_29_H_50_O. ESI-MS: 437 [M + Na]^+^; ^1^H-NMR (CDCl_3_, 600 MHz) δ_H_: 5.30 (d, *J* = 5.5 Hz, H-6), 3.51 (1H, brm, H-3α), 1.00 (3H, brs, H-19), 0.86 (3H, d, *J* = 6.0 Hz, H-29), 0.85 (3H, d, *J* = 6.5 Hz, H-27), 0.82 (3H, d, *J* = 6.2 Hz, H-26), 0.67 (3H, brs, H-18); ^13^C-NMR (CDCl_3_, 150 MHz) δ_C_: 37.33 (C-1), 31.63 (C-2), 69.51 (C-3), 41.98 (C-4), 141.17 (C-5), 119.94 (C-6), 31.15 (C-7), 31.81 (C-8), 49.57 (C-9), 36.74 (C-10), 21.66 (C-11), 39.80 (C-12), 41.98 (C-13), 55.41 (C-14), 24.19 (C-15), 28.60 (C-16), 56.04 (C-17), 11.36 (C-18), 19.30 (C-19), 36.74 (C-20), 18.75 (C-21), 33.30 (C-22), 25.73 (C-23), 45.14 (C-24), 29.15 (C-25), 20.37 (C-26), 19.30 (C-27), 23.56 (C-28), 11.03 (C-29). It was identified as β-sitosterol (Figure 4) based on the data of reference [24].

Compound **18:** Colorless oil, the molecular formula of compound **18** is C_29_H_52_O_2_. ESI-MS: 455 [M + Na]^+^; ^1^H-NMR (CDCl_3_, 600 MHz) δ_H_: 3.56 (1H, brs, H-3), 3.40 (1H, brs, H-6), 0.63 (3H, s, H-18), 0.79 (3H, s, H-19), 0.88 (3H, d, *J* = 6.4 Hz, H-21), 0.81 (6H, d, *J* = 6.6 Hz, H-26/H-27), 0.80 (3H, t, *J* = 7.8 Hz, H-29); ^13^C-NMR (CDCl_3_, 150 MHz) δ_C_: 37.3 (C-1), 31.0 (C-2), 71.3 (C-3), 32.2 (C-4), 51.7 (C-5), 69.5 (C-6), 41.7 (C-7), 34.3 (C-8), 53.8 (C-9), 36.1 (C-10), 21.1 (C-11), 39.8 (C-12), 42.5 (C-13), 56.1 (C-14), 24.2 (C-15), 28.2 (C-16), 56.1 (C-17), 12.0 (C-18), 13.4 (C-19), 36.1 (C-20), 18.7 (C-21), 33.9 (C-22), 26.0 (C-23), 45.8 (C-24), 29.1 (C-25), 19.8 (C-26), 19.0 (C-27), 23.0 (C-28), 12.0 (C-29). It was identified as 3β,6α-diol-stigmastane (Figure 4) based on the data of reference [25].

### 3.3. Nematicidal Activity of Compounds

Compounds **1, 2, 4, 5**, **6, 8,** and **9** were tested for their nematocidal activity against *M. incognita* and *P. redivivus*. The results showed that all seven compounds caused less than 15% nematode mortality at 48 h (Figure S1) when the concentration of tested compounds was 400 ppm, and also did not show significant differences compared to the control.

## 4. Discussion

Nosporin C (**1**) and nosporin D (**2**) are newly discovered metabolites in this study, and their structure types are polyketides. In previous research, nosporins A and B, which are the structural analogues of nosporins C (**1**) and D (**2**), were isolated from the filamentous fungus VKM-3750, and possessed cytotoxic effects on the sea urchin *Strongylocentrotus intermedius* and antibacterial effects on Gram-positive bacilli [10].

3-Chloro-4-methoxybenzaldehyde (**3**) was obtained from the white-rot basidiomycete *Pleurotus ostreatus*. 4-Methoxybenzaldehyde is the structural analog of **3,** which has one less chlorine substituent group than **3** and has efficacy against *Bacillus subtilis*, *Pseudomonas aeruginosa*, *Aspergillus niger*, and *Fusarium oxysporum* [26]. This compound was also discovered in *Anthracophyllum discolor*, showing antibacterial activity [27]. 3-Chloro-4-methoxybenzoic acid (**4**) was isolated from *Bjerkandera adusta*, which can promote the activity of two key protein degradation systems in human foreskin fibroblasts, the autophagy–lysosomal pathway (ALP), and the ubiquitin–proteasome pathway (UPP). It is important in the development of new regulators of the proteostasis network and has the potential to be an anti-aging agent [28]. In addition, **4** has been reported to possess anti-*Escherichia coli* and anti-*Candida albicans* activities [29]. 2-Chloro-1-methoxy-4-(methoxymethyl)benzene (**5**) is an aromatic compound [12] whose structural analog (methoxymethyl)benzene is a major constituent of the floral scents of *Nymphaea lasiophylla* and *Nymphaea lingulata* [30]. 

The organic synthesis process of 3-hydroxy-3-methyloxindole (**6**) [31] has been reported. This imine may be oxidized to **6** by a cytosolic enzyme, aldehyde oxidase [31]. The substitution and cyclization processes with indole ring-related compounds have subsequently been reported [13]. The vitamin B group’s well-known pharmaceutical compound, nicotinic acid (**7**), has garnered a lot of attention in recent years due to its crucial function in the treatment of human disorders like pellagra. This compound has anti-tuberculosis activity [32] and fibrinolytic activity [33]. In the chemical industry, succinic acid (**8**) is a highly valued biological raw ingredient. It serves as a precursor for a variety of other compounds [34], such as 1,4-butanediol, tetrahydrofuran, biodegradable polymers, and fumaric acid. In a previous study, the concentration of this compound was positively correlated with the area of colonic mucosal erosion formation in rats [35].

Uridine (**11**) is the precursor substance for uracil, which is widely produced in nature via the decarboxylation of uronic acid that is catalyzed by the enzyme uridine decarboxylase [36]. It has been shown that uracil can be used as a nutrient source in the tumor microenvironment, and studies targeting the uracil synthesis pathway suggest that uracil may become a new target for cancer and immunotherapy in the future [36]. 2′-Deoxyuridine (**12**) is a nucleoside analog that has a very similar chemical composition to uracil but lacks the 2′ hydroxyl group, which is used in antiviral medicines that are derivatives of deoxyuridine, and the application of **12** is as a precursor in the production of edoxuridine [19]. Thymidine (**13**) is also isolated from *Hydrilla verticillata* [20], and the structural analog of **13**, azidothymidine (AZT), is commonly used to treat HIV infection [37]. 1,2-Benzenedicarboxylic acid bis(2-methyl heptyl) ester (**16**) is obtained from *Phellinus linteus*, and previous in silico and in vitro results have validated that **16** could be exploited as a promising pancreatic lipase inhibitor [38]. β-Sitosterol (**17**) has been reported to be present in different parts of plants, such as fruits, leaves [39], and rhizomes [40], and possesses anti-inflammatory and immunomodulatory activities [41].

## 5. Conclusions

Nematode-trapping fungi can capture nematodes by producing traps. Some recent studies have shown that metabolites play a role in the process of these fungi capturing nematodes, such as 3-methoxy-3-methyl-1-butanol [42] and 6-methylsalicylic acid [43], with nematode attraction activity identified from *Orbilia oligospora* and *Arthrobotrys flagrans*, and C-280 [44] with nematicidal activity isolated from *O. oligospora*. These results indicate that nematode-trapping fungi have the potential to produce a variety of active secondary metabolites.

In our previous studies, the genome of *D. haptotyla* YMF1.03409 was found to contain relatively rich information on biosynthetic gene clusters, and a compound with broad-spectrum nematicidal activity, 2-furoic acid, was identified. In addition to being isolated from its fermentation products, 2-furoic acid could increase production during the process of *D. haptotyla* YMF1.03409 infection with nematodes. These results suggest that *D. haptotyla* YMF1.03409 possesses the ability to produce abundant metabolites [45]. Therefore, in this study, we continued to carry out further investigation on the metabolites of *D. haptotyla* YMF1.03409. By extracting and isolating the fermentation products, a total of eighteen compounds were purified and identified as polyketides, steroids, aromatic compounds, organic acids, and nucleosides, including two new polyketides, nosporins C (**1**) and D (**2**). Some of these compounds have also been reported to possess diverse activities in previous research. In the future, metabolic regulation can be employed to boost the active secondary metabolites of *D. haptotyla* YMF1.03409 and apply them to biological control. Alternatively, the secondary metabolites information in *D. haptotyla* YMF1.03409 could be further mined via the heterologous expression technique. Our study deepens the understanding of the secondary metabolites of *D. haptotyla* YMF1.03409 and also lays the foundation for the application of this species in the future. 

## Figures and Tables

**Table 1:** The formulation of screening media.

Media Number	Media Formulation
#1	0.5 g KH_2_PO_4_, 0.3 g MgSO_4_, 3 g yeast extract, 10 g glucose, 10 g sodium glutamate, 20 g malt extract, 20 g mannitol, 1 L H_2_O
#2	12.2 mg 5-azacytidine, 0.3 g MgSO_4_, 3 g yeast extract, 0.5 g KH_2_PO_4_, 10 g glucose, 10 g sodium glutamate, 20 g malt extract, 20 g mannitol, 1 L H_2_O
#3	4 g yeast extract, 4 g glucose, 10 g malt extract, 1 L H_2_O
#4	12.2 mg 5-azacytidine, 4 g yeast extract, 4 g glucose, 10 g malt extract, 1 L H_2_O
#5	60 g rice, 50 mL H_2_O
#6	0.3 g tryptone, 60 g rice, 50 mL H_2_O
#7	0.3 g tryptone, 60 g rice, 50 mL H_2_O, 5 g pork liver
#8	0.3 g (NH_4_)_2_SO_4_, 60 g rice, 50 mL H_2_O
#9	0.3 g (NH_4_)_2_SO_4_, 60 g rice, 50 mL H_2_O, 5 g pork liver

**Table 2:** The NMR data of compounds nosporins C (**1**) and D (**2**) (δ in ppm, *J* in Hz).

Position	1 (in CDCl_3_)			2 (in CD_3_OD)		
	^1^H	^13^C	HMBC	^1^H	^13^C	HMBC
1	-	203.4, s	-	-	209.4, s	-
2	5.35 (s)	105.1, d	C-1, C-3, C-4, C-5	5.41 (s)	106.1, d	C-1, C-3, C-4, C-5
3	-	187.8, s	-	-	191.4, s	-
4	3.28 (brs)	56.9, d	C-1, C-2, C-3, C-9, C-8	3.24 (brs)	58.5, d	C-1, C-2, C-3, C-9, C-8
5	-	58.3, s	-	-	63.1, s	-
6	3.92 (d, *J* = 9.2)	69.5, t	C-1, C-5, C-9	3.65 (d, *J* = 10.7)	70.0, t	C-1, C-9
	4.06 (d, *J* = 9.2)		C-1, C-8, C-9	3.92 (d, *J* = 10.7)		C-1, C-9
8	5.59 (s)	98.4, d	C-3, C-5, C-6, 3-OCH_3_	5.40 (brs)	99.3, d	C-3, C-5, C-6, 3-OCH_3_
9	4.34 (d, *J* = 11.0)	62.9, t	C-1, C-4, C-5, C-6, 9-OCHO	3.76 (d, *J* = 9.0)	62.9, t	C-1, C-4, C-5
	4.54 (d, *J* = 11.0)		C-1, C-4, C-5, C-6, 9-OCHO	3.81 (d, *J* = 9.0)		C-1, C-4, C-5
3-OCH_3_	3.90 (s)	59.3, q	C-3	3.93 (s)	60.2, q	C-3
9-OCHO	8.03 (s)	160.3, d	C-9	-	-	-

## Data Availability

Data are contained within the article and supplementary materials.

## Supplementary Materials

The following supporting information can be downloaded at: https://www.mdpi.com/article/10.3390/microorganisms11112693/s1, Figure S1: The nematicidal activity of compounds **1**, **2**, **4**, **5**, **6**, **8,** and **9**. (A) Activity against *M. incognita* of compounds at 400 ppm. (B) Activity against *P. redivivus* of compounds at 400 ppm.
