# Supplementary material for: Secondary Metabolites from the Nematode-Trapping Fungus Dactylellina haptotyla YMF1.03409
Source: Microorganisms. 2023 Nov 3;11(11):2693. doi: 10.3390/microorganisms11112693 (PMC10672892; doi:10.3390/microorganisms11112693)
Supplement: Supplementary file 1 [file microorganisms-11-02693-s001.zip › microorganisms-2682134-supplementary.pdf]

Supplementary materials for

Secondary metabolites from the nematode-trapping fungus *Dactylellina haptotyla* YMF1.03409

Hongmei Lei, Guangke Zhang, Peiji Zhao and Guohong Li*

State Key Laboratory for Conservation and Utilization of Bio-Resources in Yunnan, School of Life Sciences, Yunnan University, Kunming, Yunnan 650091, China

*Correspondence: ligh@ynu.edu.cn (G.L.)

Content

Figure S1. The nematicidal activity of compounds **1**, **2**, **4**, **5**, **6**, **8,** and **9**

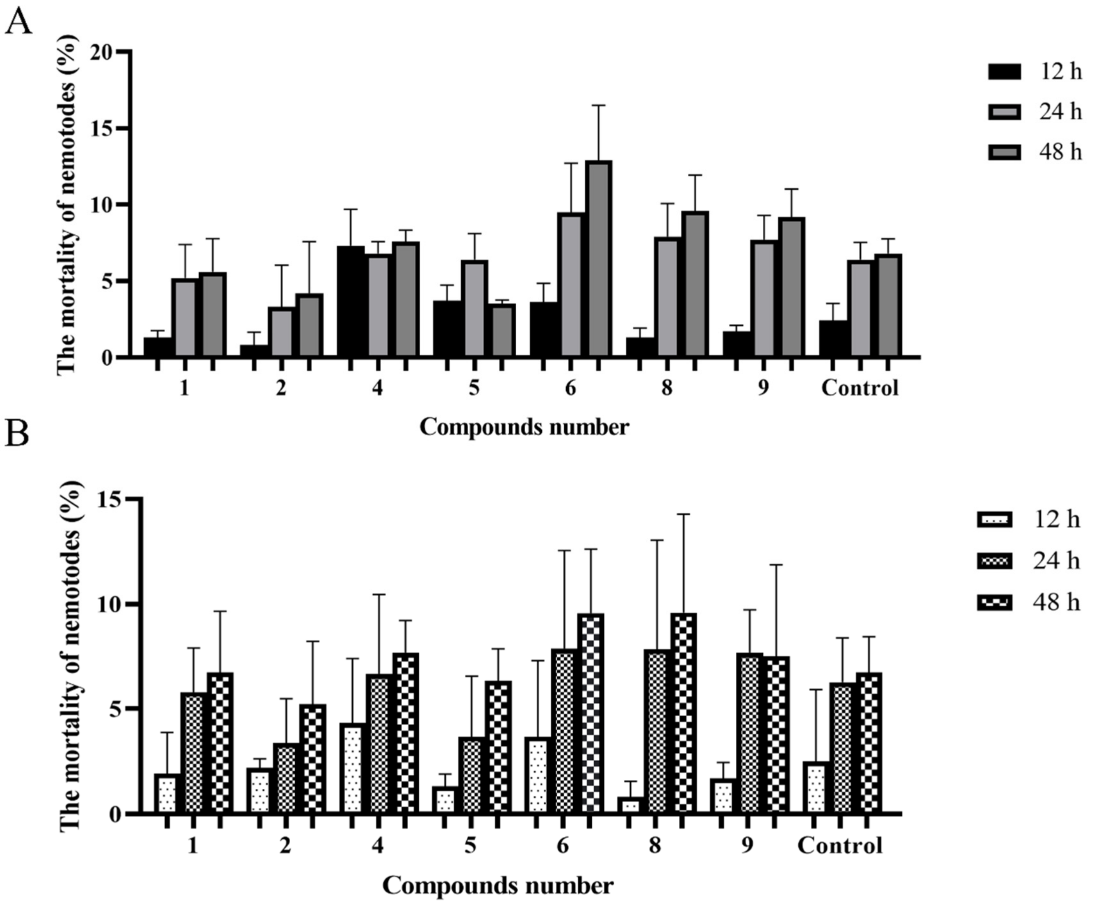

Figure S1. The nematicidal activity of compounds **1**, **2**, **4**, **5**, **6**, **8,** and **9**. (A) Activity against *M. incognita* of compounds at 400 ppm. (B) Activity against *P. redivivus* of compounds at 400 ppm.
